# Triple apheresis platelet concentrate quality after pneumatic tube system, conveyor box, and courier transport: An observational study

**DOI:** 10.1002/hsr2.596

**Published:** 2022-04-07

**Authors:** Lena Reichert, Stefan Wallner, Ralph Burkhardt, Robert Offner, Norbert Ahrens, Viola Hähnel

**Affiliations:** ^1^ Institute for Clinical Chemistry and Laboratory Medicine University Hospital Regensburg Regensburg Germany; ^2^ MVZ for Laboratory Medicine Raubling, amedes Labor Raubling Germany

**Keywords:** apheresis platelet concentrates, conveyor system, platelet function, pneumatic tube system

## Abstract

**Background and Aims:**

Platelets are prone to activation from handling; they are therefore transported as gently as possible, most commonly by courier. Speedier methods like pneumatic tube system (PTS) transport could improve patient care but may subject platelets to mechanical stress. To test the impact of mechanical stress caused by transport, we compared a PTS with a conveyor box and courier transport on apheresis platelet function.

**Methods:**

Fourteen apheresis platelet concentrate triple donations were analyzed by light transmission aggregometry (LTA), rotational thrombelastometry (ROTEM), and flow cytometry before and after indoor transport over 800 m by PTS, conveyor, and courier, respectively, while recording shocks and vibrations with a high‐frequency acceleration data logger. Shock index scores were calculated as shock intensity (g‐force) times frequency.

**Results:**

The shock index was 81 for courier, 6279 for conveyor, and 9075 for PTS. Flow cytometry revealed no significant difference in platelet surface expression of CD62p before (16%) and after transport via courier (15%), conveyor (14%), or PTS (16%). LTA with adenosine phosphate and thrombin receptor‐activating peptide‐6 resulted in comparable platelet aggregation for courier, conveyor, and PTS. ROTEM assays showed no relevant differences in coagulation time, clot formation time, and maximum clot firmness between transport modes.

**Conclusion:**

Though the mechanical challenge was smallest with courier transport, there were no significant differences in platelet activation or aggregation between the three transport modes. These data contradict restrictions on the use of PTSs for platelet concentrate transport.

## 1 INTRODUCTION

Platelets are fragile blood cells and may be activated upon mechanical stress.^1^ Activation is accompanied by shape change followed by dense and alpha granule secretion, P‐selectin (CD62p) expression, phosphatidylserine exposure, and finally membrane blebbing and microparticle formation.^2^, ^3^, ^4^

Platelet concentrates are important therapeutics in transfusion medicine to prevent or treat bleeding in patients with low platelet count or poor platelet function. Due to their fragile nature, platelets are stored in blood donation bags after collection under gentle agitation to prevent sedimentation and activation.^5^, ^6^ Taking careful handling into account, platelet concentrates are usually transported to the patient by courier or conveyor systems. Although several studies indicate that pneumatic tube system (PTS) transport does not result in a loss of platelet quality, most of them were carried out with whole blood and buffy coat‐derived concentrates instead of apheresis platelet concentrates.^7^, ^8^, ^9^, ^10^ However, considering the operating mode of PTS with its transport by air pressure, the pneumatic cushioning could prevent the cells from mechanical stress and could be appropriate for a soft and quick transport.

Therefore, this three‐arm study was designed to comparatively evaluate the impact of three different transport modes—PTS, conveyer system, and manual transport—on platelet quality and thus focused on functional tests of apheresis platelet concentrates by accelerometry, aggregometry, thrombelastography, and flow cytometry^11^, ^12^ while taking the shock load into account.

## 2 MATERIALS AND METHODS

### 2.1 Apheresis platelet collection

The study included 14 participants (all male) with a minimum platelet count of 300 × 10^9^/L in peripheral blood who donated triple platelet concentrates in autologous plasma via the Trima Accel apheresis system (Terumo BCT, software version 6), yielding a total of 42 units (collected between February and April 2020). All donors gave a written, informed consent for a research study. For ethical reasons we limited the number of donations. The platelet concentrates transported by courier and conveyor were applied for clinical use, and concentrates transported by PTS were discarded after analysis. The apheresis products were stored at 20−24°C in the connected bags with a resting time of 60–90 min, and under agitation for 60 min.^13^ Samples were drawn on the day of apheresis before separating the product into three concentrates and after transport from each bag. To draw the samples, a sterile 10 ml syringe (Product Sampling, REF: HPF0612, Cell‐Max GmbH) was used by means of the tube sealer TSCD‐II Sterile Tubing welder (Terumo BCT). The sample was injected into 3.5 ml tubes without additives (66 × 11.5 mm, SARSTEDT AG & Co. KG, REF: 60.549.001). This study protocol was approved by the local ethics committee of the University of Regensburg (19‐1516‐101).

### 2.2 Transport

The first bag of each triple donation was sent by conveyor, the second by PTS, and the third by courier over a distance equivalent to 800 m. Each transport container was equipped with a high‐frequency acceleration data logger (MSR 165; MSR Electronics). All transports and tests were performed on the same day. The conveyor (Siemens system type SimaCom VT; Telelift GmbH) is a rail transport system with special transport boxes for light freight; it transported the platelet concentrates at walking speed (0.5 m/s) and covered the transport distance in 30 min.^14^ The PTS (Sumetzberger) transported the products in cylindrical capsules by compressed air at a speed of 3 m/s and covered the transport distance in 4 min.^15^ Manual transport by courier took about 8 min.

### 2.3 Analytics

#### 2.3.1 Accelerometry

**Figure 1:**
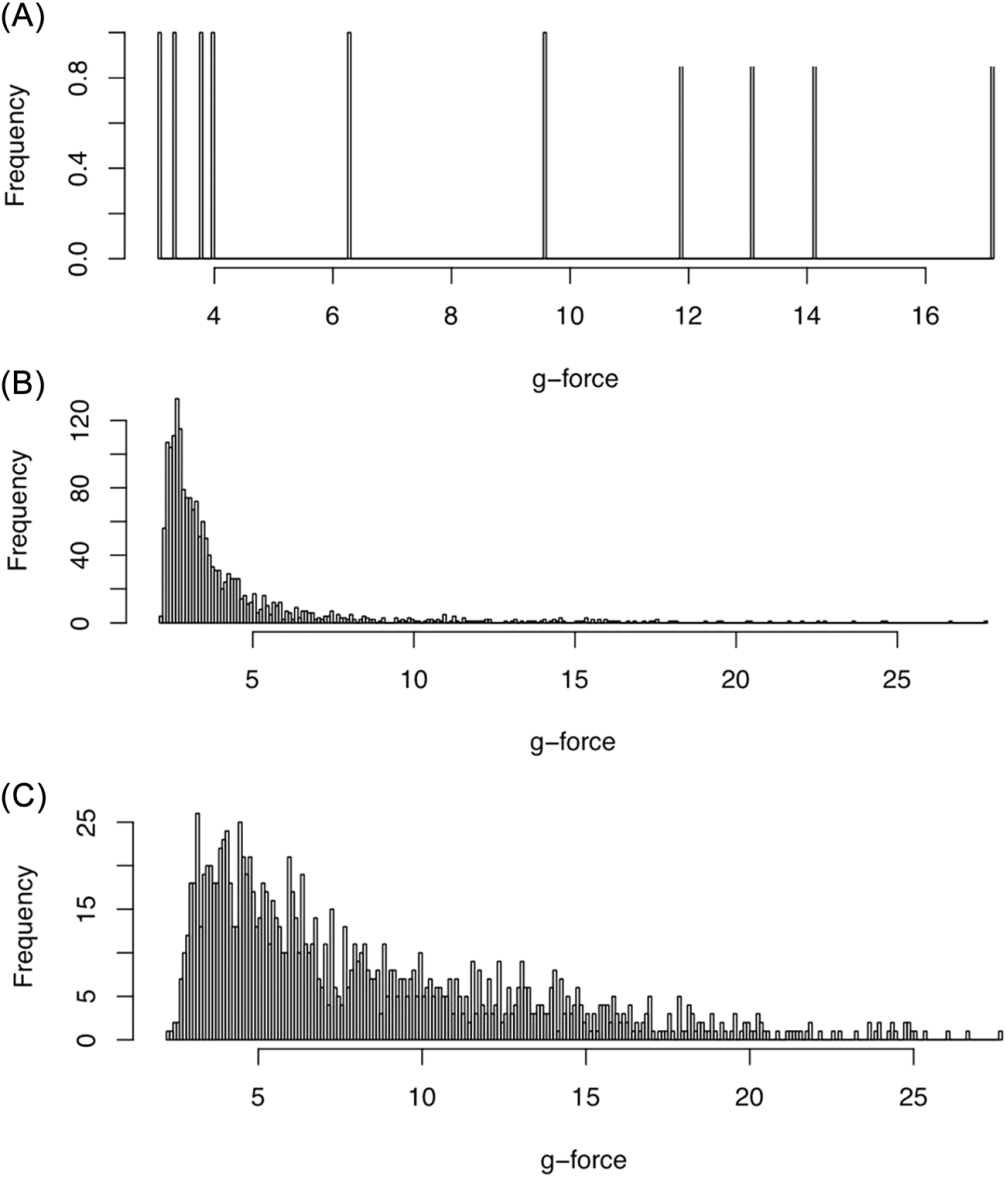
Exemplary analysis of shock intensity (g‐force) and frequency values associated with the three transport modes: (A) manual, (B) conveyor system, and (C) pneumatic tube system (PTS).

During transport, an acceleration data logger (MSR 165; MSR Electronics) measured shocks and vibrations over time (shock load) at a frequency of 800 Hz with a three‐axis acceleration sensor. Raw data were converted to.csv files and analyzed with R. G‐force measurements for each transport mode are shown in Figure 1. Based on acceleration along the *X*, *Y*, and *Z* axes, the acceleration vector sum (*A*_sum_) of a time series was calculated as the square root of the sum of acceleration along each axis squared (*_Ax_* ² + *A_y_* ² + *A_z_* ²) to express the shock load. Shock index scores were calculated as the shock intensity (g‐force) times frequency of shocks greater than the predetermined threshold of 2 g (force). The cut‐off value of 2 g was chosen in such a way that, on the one hand, the noise of small vibrations was not taken into account, and on the other hand, it was chosen so low that as many shocks as possible could still be taken into account. This was assumed at 2 g but is based on assumptions.

#### 2.3.2 Light transmission aggregometry (LTA)

LTA was performed for measurement of platelet aggregation 20 min after transport. Briefly, 300 µl samples from the transported platelet units were diluted with 300 µl physiological saline (NaCl; B. Braun). After 3 min incubation, activation was started by the following agonists: adenosine diphosphate (ADP, final concentration: 0.2 mmol/L), arachidonic acid (AA, ASPI test, final concentration: 15 mmol/L), and thrombin receptor‐activating peptide 6 (TRAP‐6; Bachem Biochemica, final concentration: 1 mmol/L). After 6 min, the maximum increase in light transmission (percent aggregation) was measured with a Multiplate Analyzer (software version V2.03.11; Roche Diagnostics) as described previously.^12^

#### 2.3.3 Flow cytometry

**Figure 2:**
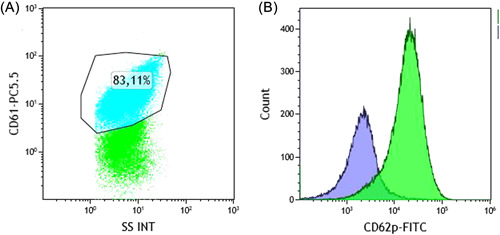
Exemplary plot (A) and overlay histogram (B) of CD62p expression from unstimulated (blue) and TRAP‐6‐stimulated (green) platelets with the mean fluorescence intensity (MFI) shift after stimulation.

Platelet staining was performed after a resting period of about 30 min. Briefly, 20,000 cells/tube were either added to phosphate‐buffered saline or stimulated with TRAP‐6 for 10 min (final concentration: 10 µM). Phenotyping of platelets was performed with commercially available CD62P‐FITC, CD41‐PE (both Beckmann Coulter), and CD61‐PerCP antibodies (BD‐Biosciences). All antibodies were titrated to obtain an optimal concentration. Flow cytometry analyses were performed with the Navios Ex flow cytometer from Beckman Coulter (software version 2.1). Activation of platelets was measured by CD62p before and after TRAP6 stimulation and expressed as a fraction of this. The basal CD62p expression was calculated from the shift of mean fluorescence intensity (MFI) of unstimulated versus stimulated CD62p‐positive platelets [%] (Figure 2). Platelet flow cytometry was performed before and after transport.

#### 2.3.4 Thromboelastography

Thromboelastography of each triple (3‐unit) donation was performed before and about 2 h after transport using the rotational thrombelastometry (ROTEM) INTEM and EXTEM assays and software version 2.6.3 (all Tem Innovations GmbH) as described previously.^16^ Briefly, 200 µl of sample was added to either INTEM reagent (Tem Innovations GmbH, composed of thromboplastin phospholipid from rabbit brain, ellagic acid, CaCl_2_, preservatives, and buffer) or EXTEM reagent (Tem Innovations GmbH, composed of tissue factor, phospholipids, CaCl_2_, preservatives and buffer) and incubated for analysis of post‐transport platelet stability. Ellagic acid‐activated coagulation was determined by INTEM, and clotting time, clot formation time, and maximum clot firmness by EXTEM (results not shown).

#### 2.3.5 Cell count

Cell concentrations were measured undiluted on an XN‐550 Automated Hematology Analyzer (Sysmex) as per the manufacturer's instructions.

#### 2.3.6 Ph analysis

The pH was measured undiluted on the blood gas analyzer ABL 90 Flex (Radiometer) as per the manufacturer's instructions.

### 2.4 Statistical analysis

Microsoft Excel 2010, IBM SPSS Statistics (version 25), and R (version 3.6.3) with its library pracma were used to collect data, generate box plots, and to determine median, mean, and standard deviation values. Normal distribution was tested with Shapiro−Wilk. Data are presented as median and range. In addition, the 95% confidence interval (CI) of the mean value was given for all data. We performed the Levene test on the assumption of variance homogeneity and a two‐sided *t* test for independent samples. *p* values below 0.05 were considered statistically significant.

## 3 RESULTS

Fourteen triple donations yielded a total of 42 units with a median volume of 249 ml and a maximum content of 8 × 10^11^ platelets per apheresis (Table 1). The maximum apheresis time was 120 min per apheresis platelet concentrate triple donation, consisting of three units each. Additionally, samples removed from each donation before transport were used as controls (*n* = 14).

**Table 1:** Characteristics of the study population and platelet concentrates before transport.

Age (years)	Total blood volume (ml)	Peripheral hemoglobin (g/dl)	Peripheral hematocrit (%)	Peripheral leukocytes (/nl)	Peripheral platelets (/nl)	Mean PC volume (ml)	Platelets PC (/nl)	pH PC	Basal CD62p PC (%)
Donor and platelet concentrate characteristics									
43	6951	13.4	40.7	5	321	246	1174	—	28.5
43	5315	14.4	42.6	6.64	349	253	979	—	19.9
28	6309	15.4	44.5	7.53	326	248	1091	7.484	15.9
26	5914	13.9	42	9.37	429	245	1233	7.446	20.8
49	6662	16.4	47.9	7.84	300	238	1071	7.314	12.0
40	5284	14.1	41.4	5.01	300	249	1072	7.254	18.5
37	6261	14.4	43.1	8.41	345	247	1289	7.573	10.8
34	6358	14.5	42.4	8.59	328	250	1143	7.415	22.3
27	5629	16.1	47	6.32	295	242	1118	7.383	14.6
46	7105	15.6	45.6	8.8	240	249	1106	7.519	11.0
40	5284	13.9	39.9	4.39	337	250	986	7.482	17.3
26	5911	15.4	45.9	8.79	371	252	1131	7.523	13.1
34	6293	14.5	42.5	8.13	325	248	1215	7.425	—
26	5817	14.2	42.1	3.93	353	255	1019	7.387	16.4
Median									
35	6087	14.7	43.5	7.13	329	249	1118	7.436	16.4

*Note*: Total blood volume was calculated after the Nadler formula. The pH analysis was performed at the end of storage from the manual transported platelet concentrate (PC).

**Figure 3:**
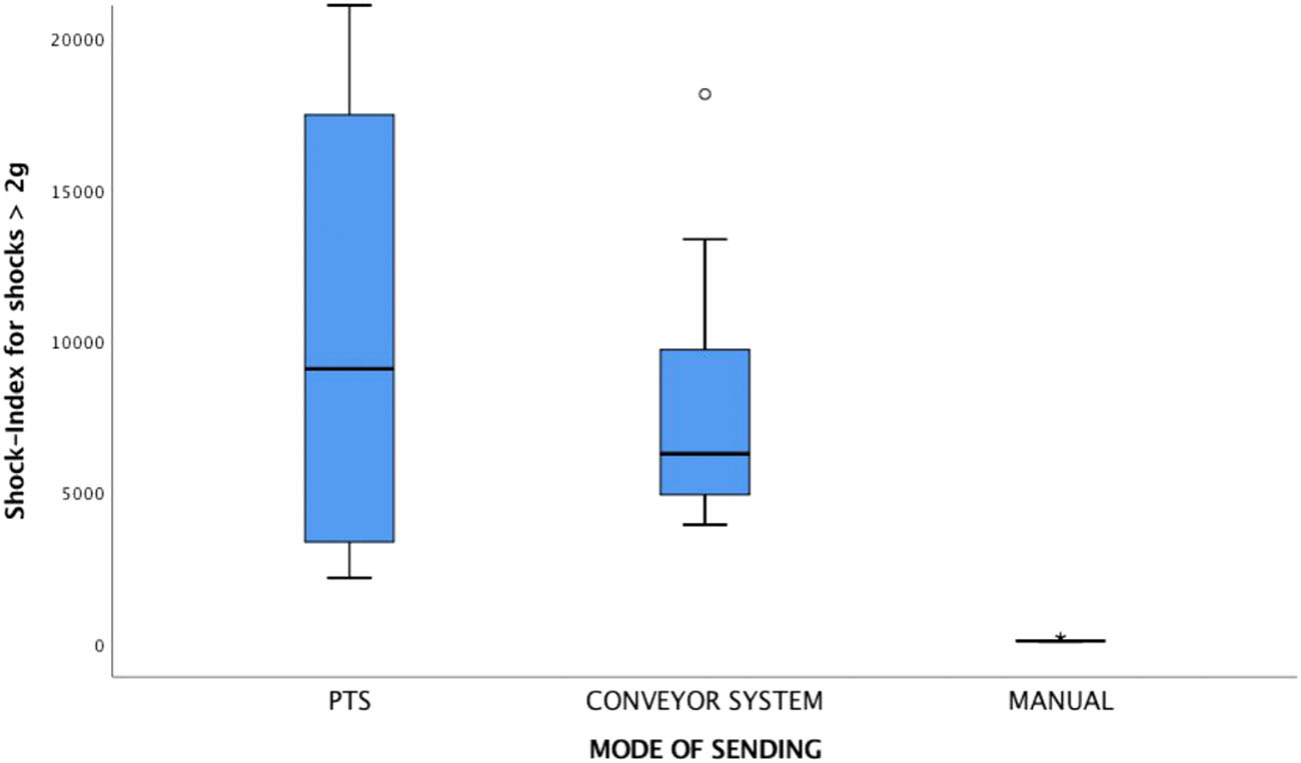
Shock index scores for shocks > 2 g (force) by transport mode.

The shock load index was 81 for courier, 6279 for conveyor, and 9075 for PTS (Figure 3). Thus, the PTS was associated with the highest dynamic mechanical load and manual transport with the lowest, while that for conveyor transport was in the same range as PTS.

Median platelet surface expression of CD62p was 16% before transport (basal), 15% after manual transport, 14% after conveyor transport, and 16% after transport with PTS (*n* = 13, see Table 2). There was no significant difference of basal CD62p expression after transport with PTS (*p* = 0.92, 95% CI: 14%−21% basal expression), conveyor (*p* = 0.07, 95% CI: 5%−49% basal expression) or manual transport (*p* = 0.95, 95% CI: 13%−19% basal expression) compared to before transport.

**Table 2:** Product data of flow cytometry, thromboelastography, and LTA are shown before and after transport.

	Before transport	Manual transport	Conveyor	PTS
Donor and platelet concentrate characteristics				
Flow cytometry				
CD62p unstimulated (MFI)	2.45 (1.80−3.85)	2.66 (1.97−3.49)	2.58 (2.16−4.65)	2.79 (2.03−3.78)
CD62p TRAP‐6 stimulated (MFI)	14.20 (9.97−21.10)	17.30 (9.54−24.00)	15.70 (3.25−24.30)	17.30 (8.76−21.90)
CD62p basal expression (%)	16.35 (10.78−28.52)	15.05 (9.75−28.93)	14.26 (10.00−143.08)	16.21 (11.26−33.79)
Thrombo‐elastography				
EXTEM CT (s)	45 (39−53)	42 (24−51)	39 (34−51)	44 (41−52)
EXTEM CFT (s)	44 (25−60)	43 (28−67)	45 (26−57)	44 (29−69)
EXTEM MCF (mm)	83 (81−89)	84 (80−88)	84 (80−88)	83 (80−88)
INTEM CT (s)	188 (163−210)	191 (171−207)	183 (156−203)	193 (175−214)
INTEM CFT (s)	26 (20−33)	26 (22−40)	24 (21−31)	26 (20−29)
INTEM MCF (mm)	85 (83−88)	84 (81−89)	86 (82−90)	84 (82−90)
LTA				
ADP (U)	68 (29−86)	62 (27−88)	66 (21−84)	64 (12−79)
ASPI (U)	89 (61−107)	91 (77−110)	92 (76−107)	90 (73−105)
TRAP‐6 (U)	75 (63−86)	76 (67−89)	77 (55−86)	76 (62−92)

*Note*: Data are presented as median values and range.

Abbreviations: ADP, adenosine diphosphate; CFT, clot formation time; CT, clotting time; LTA, light transmission aggregometry; MCF, maximum clot firmness; MFI, mean fluorescence intensity; PTS, pneumatic tube system; TRAP‐6, thrombin receptor‐activating peptide 6; U, units.

**Figure 4:**
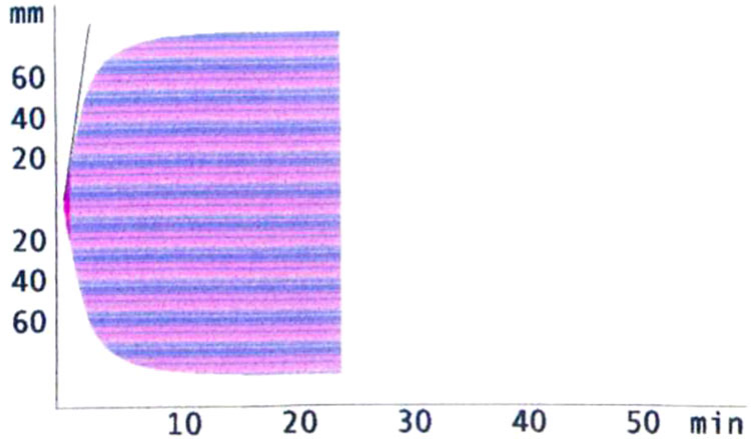
Exemplary thrombelastrogram with ROTEM EXTEM assay for pneumatic tube system. The *x*‐axis shows the time. The *y*‐axis shows the deflection.

ROTEM analysis yielded the following median clotting time, clot formation time, and maximum clot firmness values in the EXTEM test (activation of clotting by thromboplastin) for the three transport modes studied versus before transport (exemplary thrombelastogram, see Figure 4): 95%, 100%, and 101% for manual transport, 94%, 101%, and 100% for conveyor system, and 99%, 99%, and 99% for PTS, respectively. In the INTEM test (activation of coagulation via the contact phase), median clotting time, clot formation time, and maximum clot firmness values were 101%, 102%, and 99% for manual transport, 95%, 96%, and 100% for conveyor transport, and 105%, 104%, and 99% for PTS, respectively, compared to before transport. No transport mode resulted in a significant loss of quality, as indicated by these parameters, except for the conveyor system in EXTEM measurement for clotting time (*p* < 0.001, 95% CI: 89%−96%) and the PTS in INTEM measurement for clotting time (*p* = 0.03, 95% CI: 100%−106%). To guarantee accuracy, we ran four consecutive measurements per INTEM and EXTEM sample and transport mode. Accuracy was almost 100%, as was confirmed by a comparison of significance.

**Figure 5:**
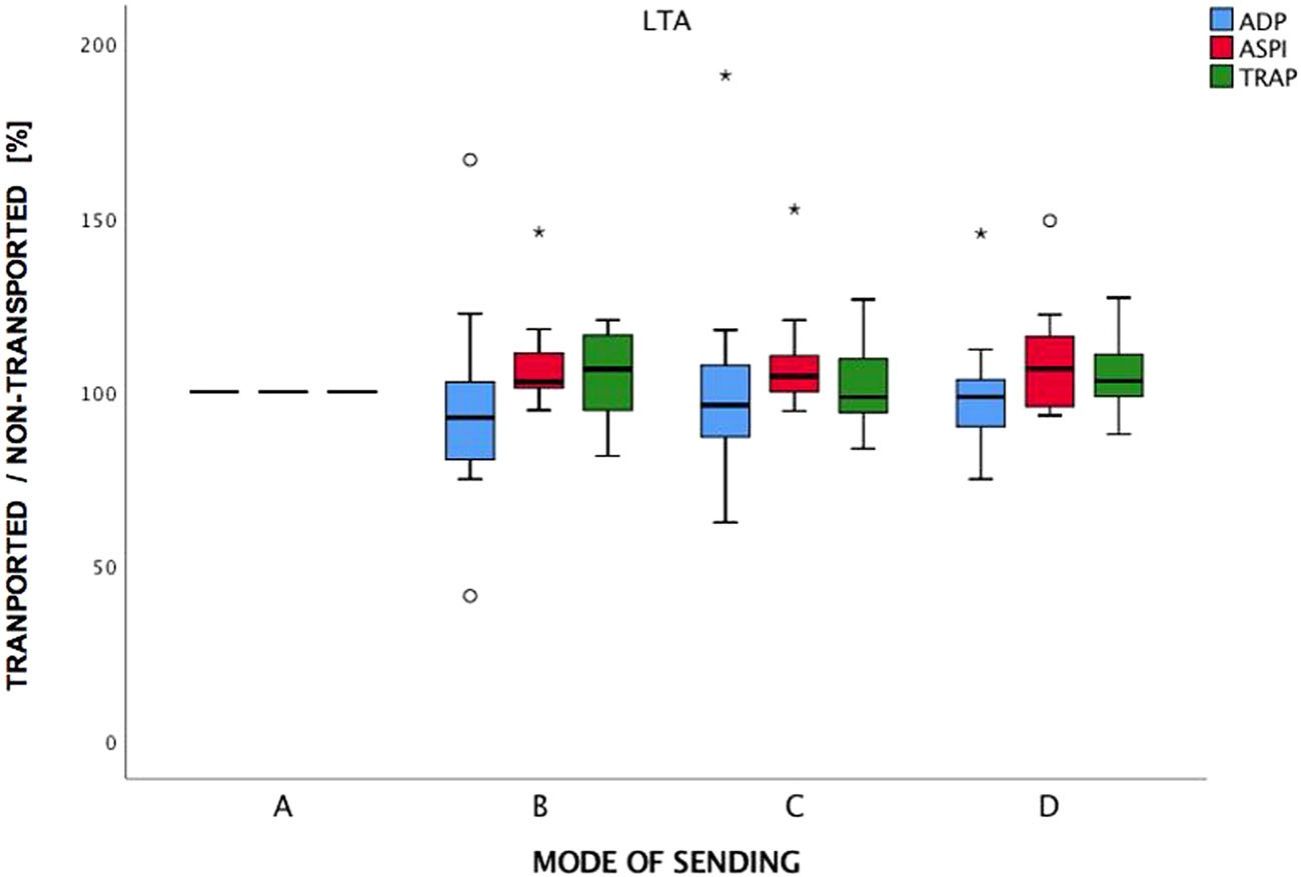
Light transmission aggregometry with adenosine diphosphate (ADP), arachidonic acid (ASPI), and thrombin receptor‐activating peptide 6 (TRAP‐6). The *x*‐axis shows the mode of transport (sending): A = non‐transported control, B = pneumatic tube system (PTS), C = conveyer system, D = manual transport. The *y*‐axis shows the results for the ratio of the mode of transport and nontransported control in per cent (%). Asterisks and small circles represent statistical significance. Outliers are marked as asterisks.

LTA with ADP, AA (ASPI test), and TRAP‐6 showed light transmission before versus after transport of 99%, 107%, and 103% for manual transport, 96%, 104%, and 98% for transport via conveyor, and 93%, 103%, and 106% via PTS (Figure 5). There was no significant loss of quality in manual, conveyor, or PTS. The only exception was in measurements using arachidonic acid as the agonist, where all three transport methods resulted in an at least partially significant change in ASPI values compared to baseline: PTS with *p* = 0.05 (95% CI: 100%−115%), conveyer with *p* = 0.05 (95% CI: 100%−117%) and courier with *p* < 0.05 (95% CI: 101%−118%). Since courier transport also resulted in a significant difference, these results could be considered comparable.

## 4 DISCUSSION

Platelet concentrates are indispensable for persons requiring platelet support, especially hematology, oncology, and surgery patients. Fast but gentle transport to the patient is essential for preserving platelet quality. The aim of our study was the evaluation of a safe transport mode. Therefore, we examined the impact of PTS, conveyor, and manual transport on platelets. The comparability of pooled and apheresis platelet concentrates is a much‐debated topic.^6^ To avoid an influence of donor variability as well as of storage,^11^ we used apheresis platelet concentrates on the day of donation. The strength of this study was that each concentrate presented its own control as each of it was split into three equal parts. Compared to conveyor transport, PTS was much faster and caused no significant loss of platelet concentrate quality, although associated with the highest dynamic mechanical load. However, the data logger recordings confirmed that manual transport subjects platelets to the lowest dynamic mechanical load. In this study, we applied LTA, flow cytometry, and thromboelastography and received information about platelet activation and coagulation. It would be of further interest to study platelet adhesion conducted by automated flow chamber systems which could give the opportunity to simulate platelet activity under physiological blood flow conditions.^17^, ^18^ This aspect was not part of our examination and limits the information given.

PTS is a popular and widely used means of transporting platelets in hospitals. Compared to manual transport, both PTS and conveyor ensure a rapid supply of patients with blood preservation and additionally save personnel by decreasing time‐consuming manual transport. However, the PTS could be susceptible to mechanical failure, defects, overuse, and abuse.^19^ Rapid acceleration and deceleration resulting in shear stress could affect platelet activation and aggregation.^20^, ^21^

Several studies considered platelet quality after pneumatic tube transport. Sandgren et al.^22^ reported the effects of transport on the quality of buffy coat platelet concentrates and figured out that it had no effect on platelet glucose consumption, lactate production, pH, pCO_2_, ATP, hypotonic shock response reactivity, CD62P, PAC‐1, platelet endothelial cell adhesion molecule 1 (PECAM1), or CD42b and caused no impairment of platelet quality. Lancé et al.^7^ investigated optical and impedance aggregation, CD62P, and microparticles in buffy coat platelet concentrates transported by pneumatic tube and identified that, first, although the number of platelets that are activated and thus become unusable for transfusion increases, this is due to storage and not to transport and, second, the most important factor influencing platelet quality is the freshness of the platelet concentrates. Transporting apheresis platelet concentrates up to three consecutive times by PTS showed no significant loss of quality regarding platelet concentration, aggregation by ADP or collagen, and CD62P expression and function. The values of some biomarkers differed significantly from the duration of platelet storage, as measurements were also performed after 3−4 days of storage.^23^ Zilberman‐Rudenko et al.^24^ sent apheresis platelet concentrates from the blood bank to the intensive care unit within their hospital by PTS and ambulatory transport (courier) while measuring gravitational force and transit time. The transported concentrates were examined in the laboratory after centrifugation. A selective loss of quality was observed.^24^ The method of centrifuging samples after transport for pelleting is controversial and was questioned the same year by Odense University Hospital in Denmark. Centrifugation had a significant effect on the quality of the platelets, so that measurements after transport and centrifugation cannot provide meaningful values about the quality by transport of the concentrates.^8^

Although further studies showed no relevant negative impact on platelet quality,^25^, ^26^ international guidelines still advise against the use of PTS transport for platelet.^27^ A recent systematic review on the impact of pneumatic tube transport on routine laboratory parameters of blood samples identified 24 relevant studies, most of which showed a correlation between PTS travel speed and distance.^28^ PTS has the advantage of shorter turnaround times, which is particularly useful in emergencies. In addition, an expansion of the systems would allow extremely long distances to be covered and logistics to be organized more efficiently. Current recommendations of the International Council for Standardization in Haematology (ICSH) cover platelet function tests prior to the use of PTS for blood samples to analyze the impact of vibration and movement on platelets.^29^

In addition, the shock load also plays a decisive role and must be considered when evaluating the influence of transport on platelet quality. Thus, all transport containers used in our study were equipped with acceleration data loggers, because, for example, the higher the acceleration, the higher the influence on the ROTEM parameters.^30^ In particular, using a three‐axis accelerometer offers the opportunity to record a combination of acceleration, magnitude of force, and duration of the transport.^31^, ^32^ However, modern PTS dispose regulated transport speed with an acceleration control.^33^ Before transporting apheresis platelet concentrates via the PTS, acceleration forces should be measured at each facility and validation using a quality threshold should be performed.^9^, ^19^, ^34^ An appropriate study design for this is provided by Garcia et al.^35^ or by Stangerup et al.^36^ who performed a 5‐day validation using mini data logger.

As transport conditions vary between locations, each hospital should individually assess the suitability of its local automated transport system. Based on the available evidence, we conclude that all three transport modes analyzed are suitable for the transport of apheresis platelet concentrates and that PTS exhibited no relevant quality loss.

## AUTHOR CONTRIBUTIONS

**Lena Reichert**: data curation; formal analysis; investigation; writing—original draft. **Stefan Wallner**: data curation; formal analysis. **Ralph Burkhardt**: resources; writing—review and editing. **Robert Offner**: writing—review and editing. **Norbert Ahrens**: conceptualization; formal analysis; project administration; supervision; writing—review and editing. **Viola Hähnel**: conceptualization; data curation; formal analysis; writing—review and editing. Open access funding enabled and organized by Projekt DEAL.

## CONFLICTS OF INTEREST

The authors declare no conflicts of interest.

## TRANSPARENCY STATEMENT

The corresponding author Viola Hähnel affirms that this manuscript is an honest, accurate, and transparent account of the study being reported; that no important aspects of the study have been omitted; and that any discrepancies from the study as planned have been explained.

## Data Availability

The authors confirm that the data supporting the findings of this study are available within this article.
